# openPDS: Protecting the Privacy of Metadata through SafeAnswers

**DOI:** 10.1371/journal.pone.0098790

**Published:** 2014-07-09

**Authors:** Yves-Alexandre de Montjoye, Erez Shmueli, Samuel S. Wang, Alex Sandy Pentland

**Affiliations:** 1 Media Lab, Massachusetts Institute of Technology, Cambridge, Massachusetts, United States of America; 2 DIG/CSAIL, Massachusetts Institute of Technology, Cambridge, Massachusetts, United States of America; University of Warwick, United Kingdom

## Abstract

The rise of smartphones and web services made possible the large-scale collection of personal metadata. Information about individuals' location, phone call logs, or web-searches, is collected and used intensively by organizations and big data researchers. Metadata has however yet to realize its full potential. Privacy and legal concerns, as well as the lack of technical solutions for personal metadata management is preventing metadata from being shared and reconciled under the control of the individual. This lack of access and control is furthermore fueling growing concerns, as it prevents individuals from understanding and managing the risks associated with the collection and use of their data. Our contribution is two-fold: (1) we describe openPDS, a personal metadata management framework that allows individuals to collect, store, and give fine-grained access to their metadata to third parties. It has been implemented in two field studies; (2) we introduce and analyze SafeAnswers, a new and practical way of protecting the privacy of metadata at an individual level. SafeAnswers turns a hard anonymization problem into a more tractable security one. It allows services to ask questions whose answers are calculated against the metadata instead of trying to anonymize individuals' metadata. The dimensionality of the data shared with the services is reduced from high-dimensional metadata to low-dimensional answers that are less likely to be re-identifiable and to contain sensitive information. These answers can then be directly shared individually or in aggregate. openPDS and SafeAnswers provide a new way of dynamically protecting personal metadata, thereby supporting the creation of smart data-driven services and data science research.

## Introduction

Personal metadata – digital information about users' location, phone call logs, or web-searches – is undoubtedly the oil of modern data-intensive science [1] and of the online economy [2]. This high-dimensional metadata is what allow apps to provide smart services and personalized experiences. From Google's search to Netflix's “movies you should really watch,” from Pandora to Amazon, metadata is used by commercial algorithms to help users become more connected, productive, and entertained. In science, this high-dimensional metadata is already used to quantify the impact of human mobility on malaria [3] or to study the link between social isolation and economic development [4].

Metadata has however yet to realize its full potential. This data is currently collected and stored by hundreds of different services and companies. Such fragmentation makes the metadata inaccessible to innovative services, researchers, and often even to the individual who generated it in the first place. On the one hand, the lack of access and control of individuals over their metadata is fueling growing concerns. This makes it very hard, if not impossible, for an individual to understand and manage the associated risks. On the other hand, privacy and legal concerns are preventing metadata from being reconciled and made broadly accessible, mainly because of concerns over the risk of re-identification [5]–[7].

Here we introduce openPDS, a field-tested personal data store (PDS) allowing users to collect, store, and give fine-grained access to their metadata to third parties. We also introduce SafeAnswers, a new and practical way of protecting the privacy of metadata through a question and answer system. Moving forward, advancements in using and mining these metadata have to evolve in parallel with considerations of control and privacy [8]–[11]. openPDS and SafeAnswers allow personal metadata to be safely shared and reconciled under the control of the individual.

### Towards Personal Data Stores

While questions of data ownership and the creation of repositories of personal data have been discussed for a long time [12]–[20], their deployment on a large-scale is a chicken-and-egg problem; users are waiting for compatible services while services are waiting for user adoption. Revelations of the collection and use of metadata by governments and companies [21]–[22] have however recently drawn attention to their potential. The combination of 1) a public interest in questions of control but also use of their data, 2) political and legal support on data ownership [23]–[26] and 3) the scale at which metadata can now be collected and processed, might trigger the large-scale deployment of PDS.

openPDS fully aligns with these trends. It uses the World Economic Forum definition of “ownership” of metadata [25]: the rights of possession, use, and disposal. It follows policies of the National Strategy for Trust Identities in Cyberspace (NSTIC) [24] and strongly aligns with the European Commission's reform of the data protection rules [23]. Finally, it recognizes that users are interacting with numerous data sources on a daily basis. Interoperability is thus not enough to achieve data ownership or address privacy concerns. Instead, openPDS implements a secure space acting as a centralized location where the user's metadata can live. openPDS can be installed on any server under the control of the individual (personal server, virtual machine, etc) or can be provided as a service (SaaS by independent software vendors or application service providers). This allows users to view and reason about their metadata and to manage fine-grained data access.

From an economic standpoint, data ownership by the individual fundamentally changes the current eco-system. It enables a fair and efficient market for metadata [2], [27] – a market where users can get the best services and algorithms for their metadata. Users can decide whether a service provides enough value for the amount of data it requests, and services can be rated and evaluated. Users are empowered to ask questions like “Is finding out the name of this song worth enough to me to give away my location?” Users can seamlessly give new services access to their past and present metadata while retaining ownership. From a business standpoint, such data ownership is likely to help foster alternatives to the current data-selling and advertising-based business model. New business models focusing on providing hardware for data collection, storage for metadata, or algorithms for better using metadata might emerge while software for data collection and data management might be mostly open-source. The proposed framework removes barriers to entry for new businesses, allowing the most innovative algorithmic companies to provide better data-powered services [2].

Other approaches have been proposed for the storage, access control, and privacy of data. Previous approaches fall into two categories: cloud storage systems and personal data repositories. First, cloud storage systems, such as the ones that have been commercially developed by companies like Dropbox [28] and Carbonite [29], are a first approximation of a user-controlled information repository for personal data. They however focus on storing files and only implement the most basic type of access control, usually on a file or folder basis. They do not suggest any data aggregation mechanisms and, once access has been granted, the raw data is exposed to the outer world, potentially compromising privacy. Second, personal data repositories have been developed in academic [12]–[17], [30], [31] and commercial settings [18]–[20]. All of these repositories are however restricted to specific queries on a particular type of data, such as interests or social security numbers. They provide only a basic access-control level, which means that once access to the data is authorized, privacy may be compromised. openPDS differs from previous approaches in its alignment with current political and legal thinking, its focus on large-scale metadata, and its SafeAnswers privacy-preserving mechanism.

### On Privacy

There is little doubt that web searches, GPS locations, and phone call logs contain sensitive private information about an individual. In 2012, 72 percent of Europeans were already concerned about the use of their personal data [23]. The recent revelations are unlikely to have helped [21], [22]. Addressing users' legitimate privacy concerns will soon be a prerequisite to any metadata usage.

Protecting the privacy of metadata is known to be a hard problem. The risks associated with high-dimensional metadata are often subtle and hard to predict and anonymizing them is known to be very hard. Over the last years, numerous works have exposed the risks of re-identification or de-anonymization of apparently anonymous datasets of metadata. An anonymous medical database was combined with a voters' list to extract the health record of the governor of Massachusetts [7] while the Kinsey Institute database was showed to be re-identifiable using demographics [32]. Twenty million web queries from around 650,000 AOL users were found to be potentially re-identifiable thanks to people's vanity searches [33] while the Netflix challenge dataset was de-anonymized using users' ratings on IMDB (The Internet Movie Database) [6]. Finally, mobility datasets of millions of users were found to be potentially re-identifiable using only four approximate spatio-temporal points [5].

Geospatial metadata, the second most recorded information by smartphone applications [34], [35], is probably the best example of the risks and rewards associated with metadata [36]. On the one hand, a recent report of the Electronic Frontier Foundation [37] worries about potentially sensitive information that can be derived from geospatial metadata. For example, geo-spatial metadata behavior collected from mobile phones has been shown to be very useful in predicting users' personalities [38]. On the other hand, the number of users of location-aware services, such as Yelp or Foursquare, are rising quickly as these services demonstrate their benefits to users.

Numerous ways of anonymizing personal data beyond the simple removal of explicit identifiers have been proposed. Similar to the original 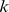-anonymity model [39], they aim minimize privacy risks while keeping data utility as high as possible. All anonymization models have however several major limitations.

Generic anonymization models have been designed for relatively low-resolution data and cannot be easily extended to high-dimensional data such as GPS location or accelerometer readings. Through generalization and suppression, 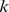-anonymity makes every record in a given table indistinguishable from at least 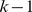 other records, thereby making it impossible to identify an individual in that table. Variations and alternatives include 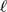-diversity [40], which address attacks based on lack of diversity in the sensitive data and 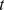-closeness [41], [42] which aims at maintaining the distribution of the sensitive data. The reader is referred to the surveys [43], [44] for further details. In metadata, any information that is unique to an individual can be used to re-identify him. Unicity (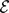) has been used to quantify the re-identifiability of a dataset [5]. Most rich metadata datasets are expected to have a high 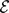. This means that, even if they are computationally tractable, generic privacy models are likely to result in most data having to be suppressed or generalized to the top-most values in order to satisfy the privacy requirement [45]. This curse of dimensionality led to the development of models dedicated to the anonymization of mobility data.

Mobility-focused anonymization models protect individual's mobility traces but only for very specific data applications or against specific re-identification attacks. The anonymization models in [46]–[50] protect the current location of the user, allowing him to anonymously perform accurate location-based searches. They however prevent any uses of historical metadata or side information, making them impractical for research and smart services using historical data. Other models [51], [52] allow for the anonymization of short successions of geospatial locations with no associated timestamps or [53] protect an individual's mobility data against re-identification at certain given times. These models however focus on anonymizing mobility data with a certain purpose or specific type of data in mind (i.e., current location, trajectory without timestamps or mobility data in given times). This makes these models impracticable for most data-science applications in academia and organizations.

Finally, all anonymization models, generic or mobility-focused, assume a setting in which the whole database is anonymized and published once. This makes it impractical, as (1) the same database is likely to be used to address different research questions (which might need specific pieces of information) and (2) smartphone applications or researchers might need access to the very latest pieces of information. Modifying existing anonymization models to support multiple releases has been shown to be non-trivial [54]. Indeed, anonymizing each publication on its own is not sufficient, since a violation of privacy may emerge as a result of joining information from different publications. Anonymizing the whole database once and successively releasing the relevant part of the anonymized data is not a solution either, since newer data may become available. Several dedicated models were recently suggested to address the multiple publications setting [54]–[57]. While very interesting, these models are based on extensions of the original one publication models and are thus very limited in the number and type of publications that they can handle.

### SafeAnswers, a new paradigm

The goal of SafeAnswers is to turn an algorithmically hard anonymization and application-specific problem into a more tractable security one by answering questions instead of releasing copies of anonymized metadata.

Under the openPDS/SafeAnswers mechanism, a piece of code would be installed inside the user's PDS. The installed code would use the sensitive raw metadata (such as raw accelerometers readings or GPS coordinates) to compute the relevant piece of information within the safe environment of the PDS. In practice, researchers and applications submit code (the question) to be run against the metadata, and only the result (the answer) is sent back to them. openPDS/SafeAnswers is similar to differential privacy [58], [59], both being online privacy-preserving systems. Differential Privacy is however designed for a centralized setting where a database contains metadata about numerous individuals and answers are aggregate across these individuals. SafeAnswers is unique, as it focuses on protecting the privacy of a single individual whose data are stored in one place by reducing the dimensionality of the metadata before it leaves the safe environment. This individual-centric setting makes it practical for mobile applications or data-science researchers. It however introduces new privacy challenges [see Analysis].

**Figure 1:**
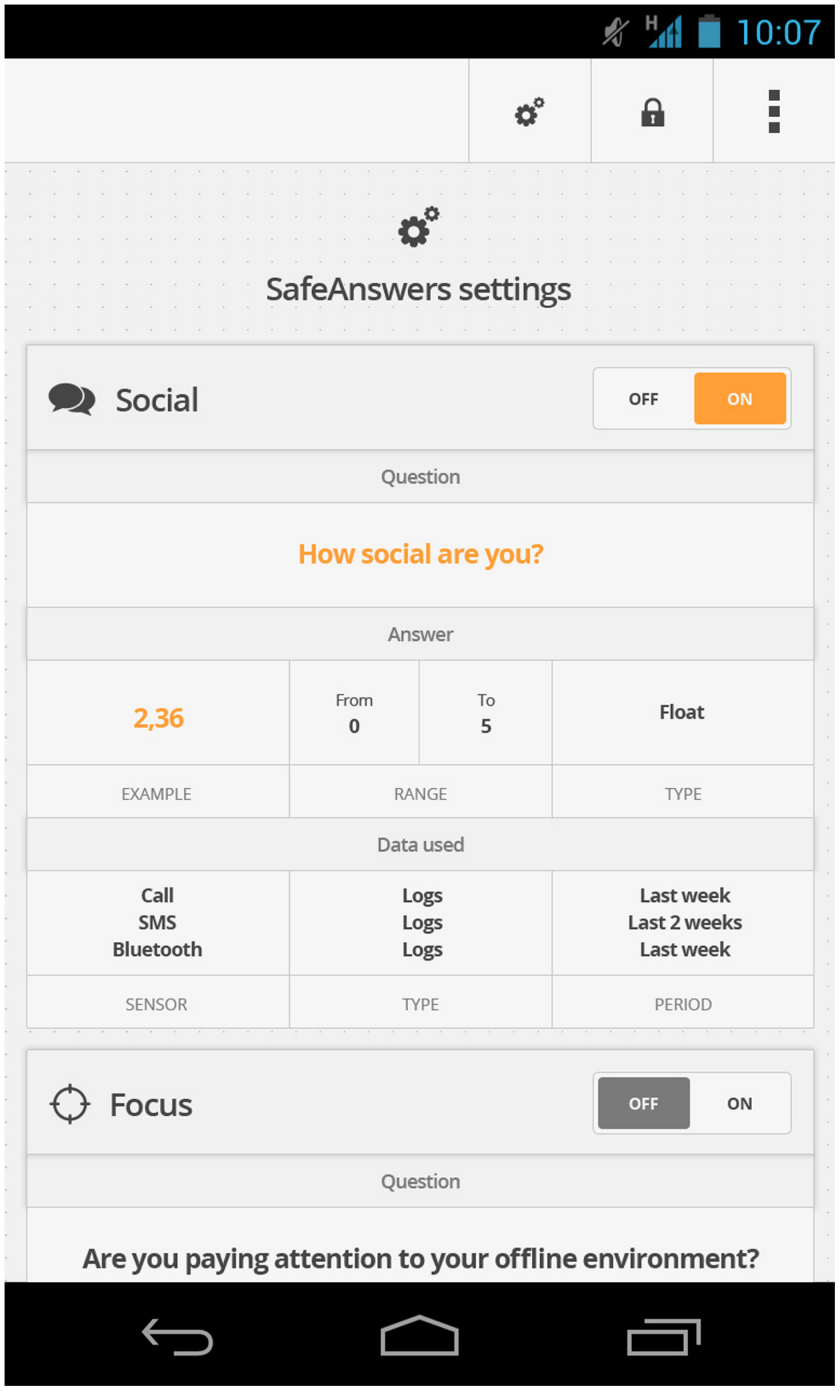
Mockups of the proposed SafeAnswers settings presented to the user for approval. This screen shows the question answered, examples of the possible responses, and the sensors used to compute the response.

**Figure 2:**
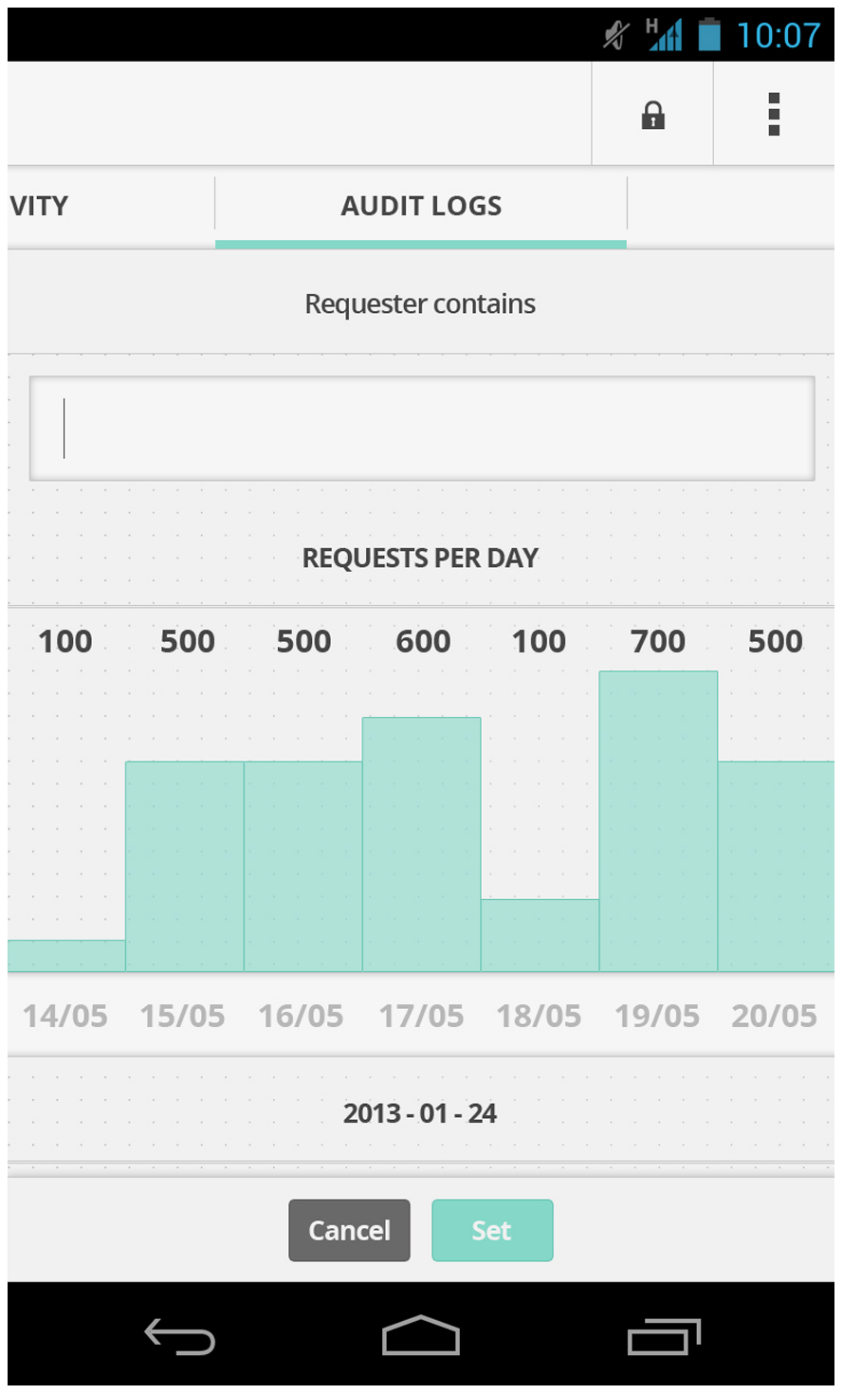
Mockups of the proposed interface showing the number of requests sent by a given app per day.

Combined with openPDS, this simple idea allows individuals to fully use their data without having to share the raw data. SafeAnswers also allows users to safely grant and revoke data access, to share data anonymously without needing a trusted third-party, and to monitor and audit data uses [Fig. 1 and 2].

## Results

### The openPDS framework

#### The Dataflow

**Figure 3:**
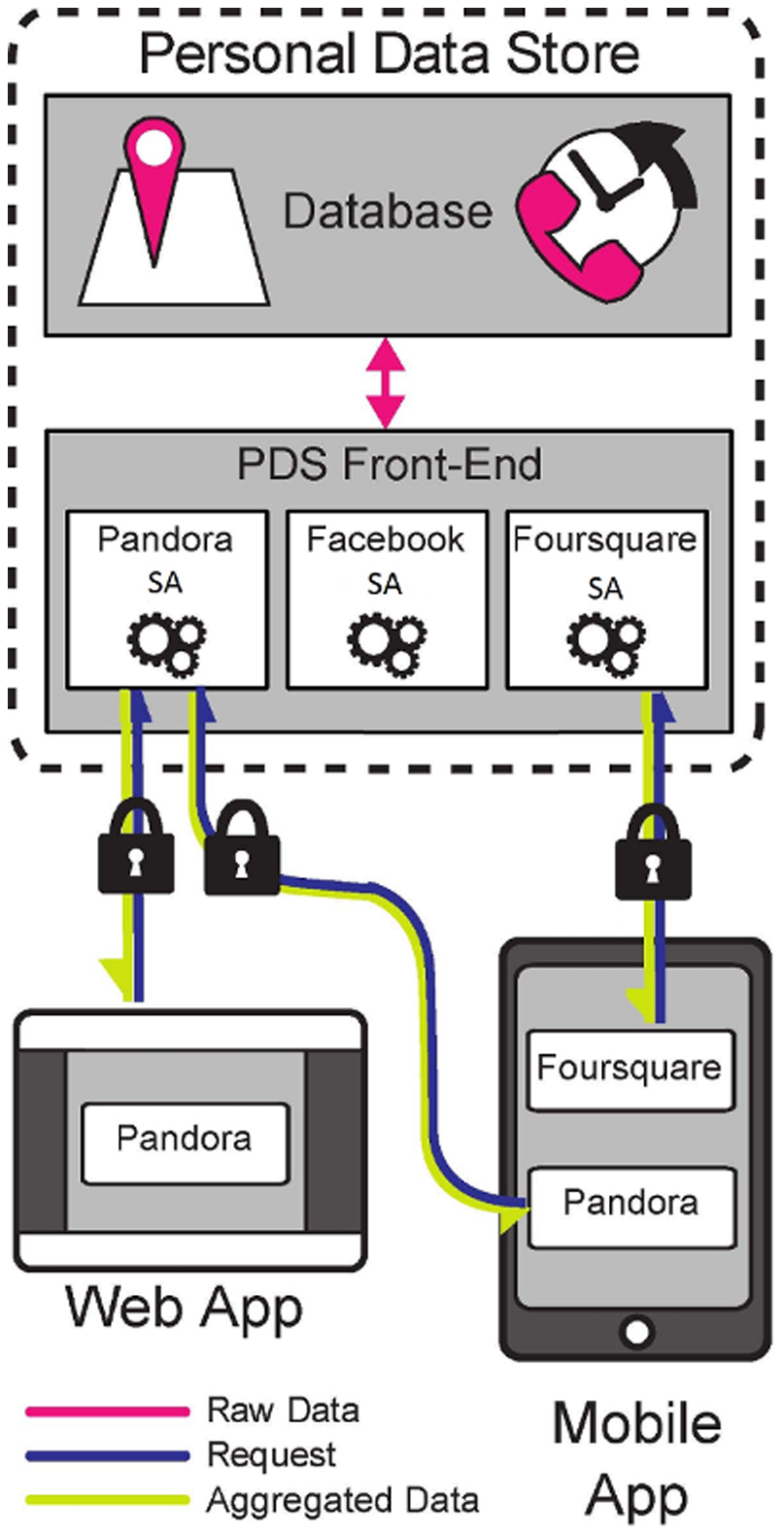
openPDS system's architecture. LBSinc web or mobile app sent a request to the user's openPDS. The request is passed on to the LBSinc SA module, which requests access to the database in order to retrieve the metadata needed to compute the answer. The SA module computes the answer, which is then validated by the PDS Front-End and send back to the web or the mobile app.

Looking at Fig. 3, consider a usecase in which a user uses a personalized music service such as PersonalizedMusic. Every time PersonalizedMusic needs to decide which song to play next on the user's mobile phone or desktop, it sends a request to the user's PDS. The actual computation of what song to play next is done by the PersonalizedMusic SafeAnswers module (SA module) inside the PDS front-end. As part of this processing, the PersonalizedMusic SA module accesses the back-end database in order to retrieve the required metadata. The PersonalizedMusic SA module would only access the raw metadata that it was authorized to when it was installed and all the processing would take place in a software sandbox. Upon completing its processing, the PersonalizedMusic SA module would return the name of the next song to play back to the front-end who will validate it and send it back to PersonalizedMusic.

#### The Database

Metadata are currently stored in a CouchDB database. CouchDB is a NoSQL store that stores data as a key to document mapping, where documents are JSON objects. CouchDB also provides a large range of existing functionality that lends itself well to the type of analysis needed to compute answers or reduce the dimensionality of the metadata. It has built-in support for MapReduce through CouchDB-Views, as well as data validation. All SafeAnswers modules share one unified database, and each SA module has a corresponding key prefix.

#### The Front-End

The front-end ensures that no unauthorized operations are carried out on the underlying metadata. SA modules are restricted to reading from the data sources they have explicitly listed as dependencies. CouchDB can also enforce access based on metadata types, time of access, time of collection, etc. The access control mechanism is implemented based on Django users and a permissioning system, where each app is registered as a user. We are working to decouple the access control mechanism and the PDS using oAuth1.0 protocol [60]. This will allow an authentication server to hand out tokens associated with a specific service and set of metadata. In addition, SA modules are executed in a sandboxed environment, and all communications are encrypted using 256 bits SSL connections. In some implementations, PDSs can be managed from a web interface.

SafeAnswers is one key innovation of the openPDS framework. SafeAnswers allows for computations on user metadata to be performed within the safe environment of the PDS. Only safe answers, the exact information needed to provide the service, leave the PDS. SA modules are intimately tied to the notion of Design Documents in CouchDB. A CouchDB design document is intended to be a document that describes an application to be built on top of an underlying CouchDB instance. Each access of the SA module to the database has to be authorized and each SA module executes inside a sandbox. We are now working to add additional fields to the CouchDB design document specification to allow additional functionality, like SA module dependencies and permissions. These descriptions will be written in the SA module manifest to be programmatically enforced and to be presented to the user before installation.

In large-scale deployments, we expect that, instead of developing a SA module from scratch for each app, there will be common libraries that can be leveraged by SA modules or directly through a standard API. For example, there could be a library that supports functionality, like returning the current city a user is in [15], his radius of gyration in the past 7 days [61] or whether he is currently running. In the future, we also hope to further develop the SafeAnswers system to support sessions. This would allow for some of the most advanced data-science uses.

### Field-studies and user feedback

Our two initial deployments offer a first qualitative evaluation of the system. The first field study is monitoring the daily behavior of individuals with diagnosed mental problems (PTSD, depression) and controls subjects for a month through their smartphones [62]. Data is used to reproduce the diagnoses of mental health conditions, focusing on changes in speech and social behavior. Recorded activities include psycho-motor activity, occupational activity, social interaction, and sleep behavior.

**Figure 4:**
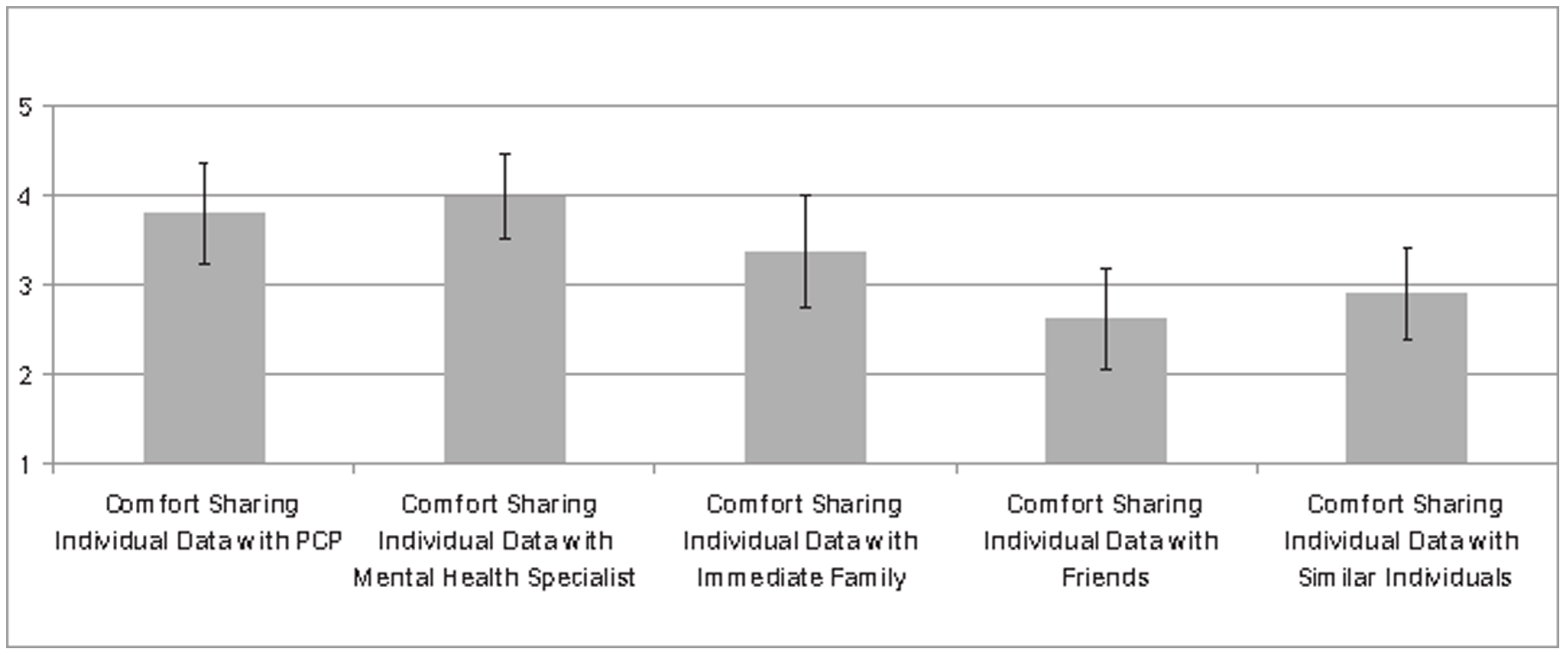
Individuals' reaction to data sharing. The error bars are bootstrapped 95% high-density intervals. We can qualitatively see that users are generally comfortable sharing individual data with their primary care provider and mental health specialist. They however seem to be less comfortable sharing such data with friends and potentially family members.

Fig. 4 presents “focus-group” results about the reaction of individuals to the openPDS framework (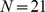, 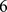 females and 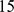 males, median age category is 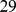 to 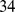 old). We consider the deployment to be a success, as 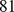
% of individuals say they would use it in their personal life and, on a 1 to 5 scale (1: “Not at all comfortable” and 5: “Extremely comfortable”), are comfortable with the data collection (mean: 4, sem: 0.27). From a privacy perspective, we can see that the ability to delete data matters to participants (mean: 4.10, sem: 0.27). We can qualitatively see that users are generally comfortable sharing individual data with their primary care provider and mental health specialist. However, they seem to be less comfortable sharing such data with friends and potentially their family members. We can also see that anonymity matters to participants (mean:4 sem:0.30) and that they are significantly more comfortable sharing anonymous, rather than individual, data (p-value <0.005 with a one-tailed, paired, non-parametric Kolmogorov-Smirnov test on 4 specific sharing questions, and mean:4 sem:0.25 when asked on the importance of anonymizing shared data). All these emphasize the relevance of the openPDS/SafeAnswers framework.

A second study, the mobile territorial lab, in partnership with Telecom Italia, Telefonica, and the Fondazione Bruno Kessler, is now underway. It is composed of 70 young parents living in Trento and its premises. The aim here is to create a long-term living lab to study user behavior and to perform user studies. Participants' behavior is recorded using an extended version of the open-sensing framework FunF [63]. All collected metadata are stored on users' PDSs.

## Discussion

### Performance

openPDS may introduce a performance overhead caused by its distributed nature, the added security and privacy mechanisms and the group computation mechanism [see Analysis].

First, the distributed nature of openPDS requires services to access the user's PDS when an answer has to be computed. In cases where computing the answer is fast, the latency it imposes might make an openPDS-based solution impracticable. Solutions such as precomputing some values and locally caching them might help. However, in cases where computing the answer inside the PDS dominates the total execution time, this might not significantly impact the user experience. In fact, this might actually introduce a performance boost, since it parallelizes the computations that are being performed at a per-PDS level.

Second, the added security and privacy mechanisms described below may also result in performance overhead. This overhead needs to be taken into account when choosing the appropriate mechanism. For example, the on-the-fly nature of openPDS/SafeAnswers may lead to inference of sensitive data if the results of several queries are joined together. On the one hand, using techniques such as the one suggested by [54] may be very efficient in preventing such inference, but they are relatively expensive in computation time. On the other hand, adding noise to query results may not be equally efficient, but would result in a much faster computation time. Advanced techniques might thus be crucial when dealing with credit card or location data, but noise addition might be sufficient to protect less sensitive data such as accelerometer readings.

For many years, group computation has been of theoretical interest only. Great improvements and actual field-studies in domains such as electronic voting, actioning, and data mining have recently made group computation–also called Secure Multiparty Computation, or SMC–of practical interest [64]. Similar to network latency, the overhead of SMC might become reasonable if computing the answer dominates the total computation time. SMC has furthermore recently been generalized into belief propagation algorithms [65]. This means that every node of the computation does not have to communicate with every other anymore, thereby reducing the overhead.

### Usage Experience

In this section we describe two short scenarios for a user and a developer switching to an openPDS/SafeAnswers system for mobile applications.

#### End-User

Suppose Alice wants to install and use a smartphone app like LBSinc, a location-based check-in application, without using a PDS. Alice downloads the app onto her phone, authorizes LBSinc to access her phone's network communication and GPS coordinates, and creates a user account with LBSinc. The LBSinc app starts collecting metadata about her and stores it all in its back-end servers. Under this model it is difficult for Alice to access the metadata LBSinc uses to makes inferences about her, or to remove the metadata she does not want LBSinc to access or use.

Alternatively, Alice could decide to download a PDS-aware version of LBSinc. She installs it just like she would install any other smartphone app and authorizes it to access only her phone's network communication. When used for the first time, the smartphone app prompts her to enter her PDS URI. Alice then sees exactly what metadata the LBSinc SA module will have access to and examples of the answers [see Fig. 2], the relevant summarized information that will be send back to LBSinc. If she accepts, the LBSinc SA module is installed onto her PDS and she can start using it.

#### App Developer

Suppose a developer now wants to implement MyMusic, a smartphone app that plays music to Alice based on her preferences and current activity. Under the current model, he would first have to develop a smartphone app to collect the metadata on Alice's phone, record it, and periodically send it to a server. He would then develop a server with an internal database to store the raw activity data he collects, a secured API for this database to receive the metadata, and a way to anonymize the metadata or at least separate the user account information from the metadata. He could then start developing an algorithm to decide which song or type of music to play. The initial picture he would have of users would be very rough, as he would have no prior metadata to work with. Finally, he would have to wait to collect a sufficient amount of metadata before being able to provide adequate recommendations.

If operating within the openPDS/SafeAnswers framework, the metadata that the developer needs are likely to have already been collected either by a metadata collection app [66] or by another application or service. The developer would then spend most of his time writing an SA module that would decide which song or type of music to play and test it on development copies of PDSs. The PDS front-end would take care of creating the API and of securing the connection for him. The developer's algorithm would be able to access a potentially large set of metadata, including historical metadata.

## Analysis

The openPDS framework suggests several mechanisms for enhancing the privacy and security of personal metadata: SafeAnswers, access control, sandboxes, and network encryption. In this section, we discuss several cases where these might fall short and discuss potential counter-measures.

### Protecting aggregated answers of groups

A practical example would be a service, such as CouponInc, which wants to execute a simple query to know how many of its users are around a certain shop to send them a special coupon. CouponInc might want to issue a query like “How many users are in this geographical area at the current time?” or “How active are these users during lunch time?”

In a distributed setting, such computation falls under the well-studied field of secure multi-party computation (SMC) [67], where the querying agent never sees any individual user's metadata but can access information aggregated across users. User privacy is preserved, as each PDS only sends cryptographically masked messages to other nodes in the network.

**Figure 5:**
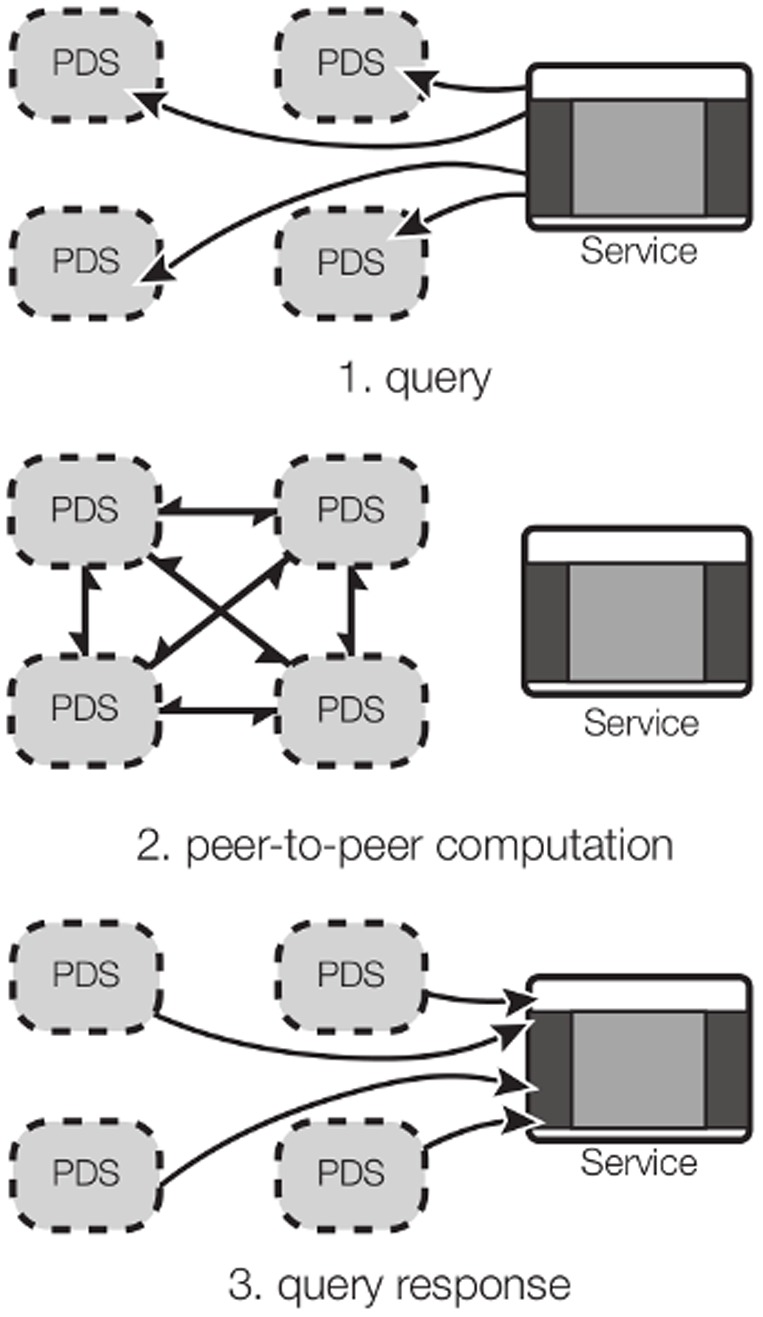
Group Computation Overview. (1) A querying agent (like CouponInc) passes a function that its wants a collaborative answer for, along with a list of URI to PDSs. (2) PDSs all trade messages in order to compute a collaborative answer. (3) The answer is reported back to the querying agent.

Such a cryptographic technique fits elegantly into the PDS model of computation [Fig. 5]. Rather than anonymizing and computing over-complex data items, like GPS coordinates, the SA modules could compute features locally to each user's PDS, reducing the dimensionality of the metadata. After the local computation is done, the inferred facts–e.g. whether or not a given user is in a given geographical area–can be aggregated in a privacy-preserving way. This means that even the low-dimension answer cannot be associated with a particular user.

### Attacks in the case of well-behaved apps

Even in the absence of attackers, apps that behave as they are supposed to might pose a risk to users' privacy. We notice two major challenges: (1) How can an openPDS/SafeAnswers determine the required level of aggregation given that it only has access to the metadata of a single user? (2) Well-behaved apps could inadvertently collect data whose combinations may allow others to infer sensitive information.

A potential solution to the first challenge might be found in [5]. The authors studied fifteen months of human mobility data for one and a half million individuals, and found that one formula determines the uniqueness of an individual's mobility traces, given the traces' resolution (i.e., level of aggregation) and the amount of background knowledge available to the adversary. If extended to other types of data, such an equation could be used by SafeAnswers to determine the required level of aggregation needed when answering a query.

The fields of Privacy Preserving Data Publishing and Mining aim to address a problem similar to the second challenge: how to anonymize the current publication of a database so that the combination of all past and current anonymized publications respect privacy. These works suggest several interesting assumptions and techniques that could be adopted by the openPDS/SafeAnswers framework. For example, the authors of [54] show that the problem of accurately calculating the level of privacy imposed by a set of three or more publications is NP-hard. The authors then suggest a relaxed method for calculating the privacy level in polynomial time. Their method is based on joining the set of publications into a single table, which can then be checked against some privacy requirement. They also suggest a supplementing algorithm for anonymizing the current publication so that the required privacy level is obtained. Their methods might be used by SafeAnswers in order to determine whether the current set of queries and potential future queries might compromise privacy.

Work in statistical databases might also help address the second challenge [68]. A statistical database aims to allow the execution of statistical queries without compromising the confidentiality of any individual represented in the database. Two approaches used in this field could be useful for SafeAnswers: (1) A query restriction rejects each query that could compromise a user's privacy and provides accurate answers to legitimate queries. The computation of what is a legitimate query is usually based on the size of the query's results or the extent of overlap between queries. Note however that the denial of a query may, in itself, provide information to an an attacker. (2) Perturbation gives approximate answers by adding noise to the answers computed from the original metadata. Regardless of the specific perturbation technique, the designer must attempt to produce statistics that accurately reflect the underlying database. Such perturbed answers might however not be acceptable for all uses.

### Attacks in the case of malicious apps

While well-behaved apps might inadvertently collect sensitive information, apps that are voluntarily not playing by the rules pose a serious threat to user's privacy. The major risk we see here is how to protect the metadata against an app that deliberately tries to infer sensitive information by over-querying a user's openPDS or by colluding with other apps.

Technically, numerous techniques from anomaly detection may help SafeAnswers detect suspicious behavior. For example, a service that suddenly changes its query pattern; querying for location every minute while it used to ask user's location and speed a few times in a row 3 times a day. The detection of anomalies, outliers, or rare events, has recently gained a lot of attention in many security domains, ranging from video surveillance and security systems to intrusion detection and fraudulent transactions. Accordingly [69], most anomaly detection methods are based on the following techniques: classification, nearest neighbor, clustering, statistical, information theoretic, and spectral. Any of these techniques, or their combination, can potentially be used by SafeAnswers.

Anomaly detection could also be combined with reputation systems to allow for a group of openPDSs to exchange information about modules and services in real-time. The P2P reputation systems literature considers different types of malicious behavior that can be blocked with the help of reputation systems. These give us a foretaste of potential risks. “Traitors” are services who initially behave properly but then start to misbehave and inflict damage on the community. This technique is particularly effective when the service has become respectable and well installed. “Whitewashers” are services who leave and rejoin the system with new identities in order to purge the bad reputations they acquired under their previous identities. Finally, “Collusions” are a group of malicious services acting together to cause damage. Such reputation systems could be combined with other privacy mechanisms discussed here. For example, an openPDS might decide to allow a service with a medium rating to execute restricted or noisy queries but temporarily block a service whose rating suddenly dropped.

Various UI mechanisms can also be used to warn users of potentially malicious apps before they are installed. For example, trust could be used to rate service providers. Adapting the definition from [70], trust would reflects a user's or a PDS's subjective view of a service, while reputation could be considered a collective measure of trust reflecting a group view of that service. Work by [71] shows that the reputation of the service provider matters more than the specific data being accessed and hints at the potential usefulness of a reputation system to help users decide which services to trust. Various principles for computing reputation and trust can be found in [72]. Besides a simple summation or average of ratings, the authors mention discrete models in which trust is a discrete value from a predefined set of values, fuzzy models, bayesian systems, belief models, and flow models.

### Attacks compromising the host

Finally, openPDS is vulnerable to the traditional security and privacy issues of any hosted system. Attackers could compromise the authentication/control mechanisms or impersonate existing users to gain access to the database or to corrupt the system. For instance, in the case of virtual machines hosting openPDSs, an attacker's virtual machine can legitimately be located in the same physical machine as openPDSs virtual machines. This is, however, not specific to openPDS, and similar issues exist with any hosted systems, such as SaaS, virtual machine and traditional servers. Solutions include hypervisors [73] or data-at-rest encryption [74], [75] such as homomorphic encryption schemes [76]. The main difference openPDS introduces is having the data distributed across machines, systems, and implementations of openPDS. While a full analysis is beyond the scope of this paper, one might imagine that a distributed and heterogeneous system might be harder to attack than some of the traditional centralized ones especially if information is shared across machines [see previous section].

## Conclusion

Finally, as technologists and scientists, we are convinced that there is an amazing potential in personal metadata, but also that benefits should be balanced with risks. By reducing the dimensionality of the metadata on-the-fly and actively protecting users, openPDS/SafeAnswers opens up a new way for individuals to regain control over their privacy.

openPDS/SafeAnswers however still face a number of challenges. Each challenges includes several potential directions for future research: (1) the automatic or semi-automatic validation of the processing done by a PDS module; (2) the development of SafeAnswers privacy-preserving techniques at an individual level for high-dimensional and ever-evolving data (mobility data, accelerometer readings, etc.) based on existing anomaly detection framework and potentially stored in highly-decentralized systems; (3) the development or adaptation of privacy preserving data-mining algorithms to an ecosystem consisting of distributed PDSs; and (4) UIs allowing the user to better understand the risks associated with large-scale metadata and to monitor and visualize the metadata used by applications.
